# Cholera Incidence and Mortality in Sub-Saharan African Sites during Multi-country Surveillance

**DOI:** 10.1371/journal.pntd.0004679

**Published:** 2016-05-17

**Authors:** Delphine Sauvageot, Berthe-Marie Njanpop-Lafourcade, Laurent Akilimali, Jean-Claude Anne, Pawou Bidjada, Didier Bompangue, Godfrey Bwire, Daouda Coulibaly, Liliana Dengo-Baloi, Mireille Dosso, Christopher Garimoi Orach, Dorteia Inguane, Atek Kagirita, Adele Kacou-N’Douba, Sakoba Keita, Abiba Kere Banla, Yao Jean-Pierre Kouame, Dadja Essoya Landoh, Jose Paulo Langa, Issa Makumbi, Berthe Miwanda, Muggaga Malimbo, Guy Mutombo, Annie Mutombo, Emilienne Niamke NGuetta, Mamadou Saliou, Veronique Sarr, Raphael Kakongo Senga, Fode Sory, Cynthia Sema, Ouyi Valentin Tante, Bradford D. Gessner, Martin A. Mengel

**Affiliations:** 1 Agence de Medecine Preventive, Paris, France; 2 Ministère de la santé, Kinshasa, Republique Democratique du Congo; 3 Institut Pasteur, Abidjan, Cote d’Ivoire; 4 Institut National d’Hygiene, Lome, Togo; 5 Universite de Kinshasa, Kinshasa, Republique Democratique du Congo; 6 Ministry of Health, Kampala, Uganda; 7 Institut National d’hygiene Publique, Abidjan, Ivory Coast; 8 Instituto Nacional de Saude, Maputo, Mozambique; 9 Makerere School of Public Health, Kampala, Uganda; 10 Central Public Health Laboratory, Ministry of Health, Kampala, Uganda; 11 Ministere de la sante publique et de l’hygiene publique, Conakry, Guinea; 12 Ministere de la sante, Lome, Togo; 13 Institut National de Recherche Biomedicale, Kinshasa, Republique Democratique du Congo; 14 Ministere de la santé, Division Provinciale de la santé, Goma, Republique Democratique du Congo; 15 Institut National de Sante Publique, Conakry, Guinea; 16 Ami-labo, Goma, Republique Democratique du Congo; Massachusetts General Hospital, UNITED STATES

## Abstract

**Background:**

Cholera burden in Africa remains unknown, often because of weak national surveillance systems. We analyzed data from the African Cholera Surveillance Network (www.africhol.org).

**Methods/ Principal findings:**

During June 2011–December 2013, we conducted enhanced surveillance in seven zones and four outbreak sites in Togo, the Democratic Republic of Congo (DRC), Guinea, Uganda, Mozambique and Cote d’Ivoire. All health facilities treating cholera cases were included. Cholera incidences were calculated using culture-confirmed cholera cases and culture-confirmed cholera cases corrected for lack of culture testing usually due to overwhelmed health systems and imperfect test sensitivity. Of 13,377 reported suspected cases, 34% occurred in Conakry, Guinea, 47% in Goma, DRC, and 19% in the remaining sites. From 0–40% of suspected cases were aged under five years and from 0.3–86% had rice water stools. Within surveillance zones, 0–37% of suspected cases had confirmed cholera compared to 27–38% during outbreaks. Annual confirmed incidence per 10,000 population was <0.5 in surveillance zones, except Goma where it was 4.6. Goma and Conakry had corrected incidences of 20.2 and 5.8 respectively, while the other zones a median of 0.3. During outbreaks, corrected incidence varied from 2.6 to 13.0. Case fatality ratios ranged from 0–10% (median, 1%) by country.

**Conclusions/Significance:**

Across different African epidemiological contexts, substantial variation occurred in cholera incidence, age distribution, clinical presentation, culture confirmation, and testing frequency. These results can help guide preventive activities, including vaccine use.

## Author Summary

Cholera burden in Africa remains unknown, often because of weak national surveillance systems. Reporting is non-exhaustive for various reasons, such as individual and community fears of stigmatization and economic loss. Furthermore, only 3% to 5% of all cases are laboratory confirmed. A variety of case definitions are used across countries, which could lead to cholera over or under-reporting. Our study presents the first data from prospective multi-country cholera surveillance in Africa, and the only such data based on culture confirmation and that includes a description of clinical presentation. We show how confirmed cholera cases varied over time by setting, and identified three epidemiological patterns that can guide decision-making processes. We documented that reliance on suspected cases–as is usually done in national surveillance–rather than confirmed cases can over-estimate substantially cholera incidence. Finally, our surveillance strategy of using case-based reporting and a standard comprehensive case reporting form provided more information on at-risk populations and geographical hot spots than is currently available in the literature; this is turn should facilitate development of efficient preventive strategies.

## Introduction

Although cholera has disappeared as a public-health problem in developed countries, it remains a major concern in sub-Sahara Africa [1,2]. From 2007 to 2012, at least 20 African countries reported more than 100,000 cases of cholera (World Health Organization (WHO) weekly epidemiological records, 2007–2012). However, this surveillance has weaknesses. Reporting is non-exhaustive for various reasons such as individual and community fears of stigmatization and economic loss. Reporting from district to national levels may be delayed or incomplete. According to WHO, only 3% to 5% of all cases are laboratory confirmed [3]. A variety of case definitions are used across countries, which could lead to cholera over or under-reporting. Finally, few countries have implemented case-based surveillance, with information at national level provided in the form of weekly summaries limited to cumulative case numbers and deaths [1].

Since the Haiti epidemic during 2010, public and political attention on cholera has increased. Recently, WHO has prequalified a two-dose oral cholera vaccine (OCV) that is less expensive and less cumbersome to deliver than its predecessor. This, and the creation by WHO of a cholera vaccine stockpile for epidemic and potentially endemic cholera prevention, have stimulated interest in more timely, accurate, and comprehensive disease burden data from affected countries.

The African Cholera Surveillance Network (Africhol) was launched in 2009. Originally implemented in eight of the most affected sub-Saharan African countries, it has since expanded to three additional countries. Its primary aim is to better define cholera burden, geographic distribution, seasonal patterns, and risk groups to inform prevention strategies, including immunization. We present here incidence results and the associated case fatality ratio from eleven geographical areas located in the six Africhol countries having the strongest performing surveillance systems.

## Methods

### Study design

Starting in 2011, we implemented population-based cholera surveillance in all cholera treatment facilities in a given geographic zone chosen in collaboration with ministries of health (MoHs). Criteria for zone selection included: yearly occurrence of outbreaks or sporadic cholera cases; existence of dedicated diarrhea or cholera treatment facilities; and laboratory capacity for cholera confirmation by stool culture. In these zones, all health facilities providing treatment for severe cholera cases were included in surveillance. We also conducted a prospective surveillance in several outbreak sites outside of surveillance zones when these were reported to the MoH and when they had adequate laboratory facilities available. This was conducted the time of the epidemic.

### Cholera case definition

Patients were followed in all the cholera treatment facilities of a given surveillance area. In areas without known ongoing cholera, a suspected cholera case was defined as a patient aged two years or more that developed severe dehydration or died from acute watery diarrhoea. In areas with known cholera, a suspected case was defined as a patient aged two years or more that developed acute watery diarrhoea, with or without vomiting. A confirmed case was defined as a suspect cholera having a stool culture positive for *Vibrio cholerae*.

### Participating countries, surveillance zones and starting date

Eight enhanced surveillance zones located in areas of known recent cholera occurrence were included in the analysis. Their location and starting dates were as follows: 1) Togo: five districts of Lome and Golfe district, Jun 2011; 2) Togo: Lake district in the Maritime region, Jun 2011; 3) Democratic Republic of Congo (DRC): Goma and Karisimbi districts, Aug 2011; 4) Guinea: five districts of Conakry, Jul 2011; 5) Uganda: Manafwa, Mbale, and Butaleja districts, Dec 2011; 6) Mozambique: Beira city, Aug 2011; 7) Cote d’Ivoire: one district of Abidjan, Koumassi–Port Bouet–Vridi district (KPBV), Jun 2012. While data collection is currently ongoing, here we include only surveillance data collected through Dec 31^st^, 2013. In addition to surveillance zones, we included data collected during outbreaks in Kasese district, Uganda (Oct 2011–Dec 2012); Pemba city, Mozambique (Jan 2013–Dec 2013); Adiake prefecture, Cote d’Ivoire (May–Oct 2012); and three districts of Kinshasa (Maluku, Kingabwa, and Massina districts), DRC (Jul 2011–Feb 2012). Within specifically defined study zones, we included all health care facilities known to treat cholera cases, including long-term facilities as well as newly established cholera treatment centers (in Africa, these centers frequently are opened only in response to an outbreak). While all cholera cases were supposed to have been referred to a designated cholera treatment center, it is likely that private health centers conducted unauthorized evaluation and treatment. Included centers were the following: 1) Conakry, Guinea. The infectious disease and paediatric departments of Donka hospital. The additional cholera treatment center (CTC) in the Ratoma neighbourhood opened during the 2012 epidemic was also included; 2) Lome, Togo. The infectious disease and paediatric departments of the Centre Hospitalier Universitaire, Be Hospital, and other district health centres in which a temporary cholera treatment center was opened; 3) Lake District, Togo. The infectious disease and paediatric departments of Aneho Hospital and health centres with temporary treatment centers; 4) Goma-Karisimbi district, DRC: The cholera treatment centers located in the General Provincial Hospital, the Buhimba cholera treatment and the Kiziba temporary cholera treatment unit; 5) Maluku-Kingabwa-Massina district, Kinshasa, DRC: cholera treatment centers of Kingabwa and Malaku and the cholera treatment unit of Massina; 6) Abidjan, Koumassi-Port Bouet, Vridi District, Cote d’Ivoire. The infectious disease and paediatric departments of Port Bouet and Koumassi Hospitals and the temporary cholera treatment center at the Vridi Health Centre; 7) Adiake prefecture, Cote d’Ivoire: Adiake general hospital and temporary treatment centers; 8) Mbale-Manafwa-Buteleja district, Uganda: Nabiganda health center, Namatela health center and Busiu health center; 9) Kasese district, Uganda: Bwera hospital, Kayangi health center, Kagando hospital, Kinyamaseke health center and Kitholhu health center and other temporary treatment centers; 10) Beira, Mozambique: Ponta-Gea health center, Macurrungo health center, Munhava health center, Macurrungo and the central hospital of Beira; 11) Pemba city, Mozambique: the temporary cholera treatment center of Pemba city.

### Data sources and data collection

In the enhanced surveillance zones and outbreak sites, the MoH teams collected data at health centers level using the same standardized data collection forms, which included sex, age, location, date of symptoms, culture results but also clinical information such as watery diarrhea, rice water stool, vomiting, dehydration. We identified all deaths among patients admitted to a cholera treatment facility. We did not include deaths occurring in the community or after treatment center discharge. In parallel, the MoH continued to register the overall number of suspected cases in their routine surveillance system using line lists with a limited number of variables (date of onset, district, age and sex). We used district–level population estimates for 2011 or 2012 that corresponded to the geographic area under surveillance. The 2011 and 2012 population estimates were derived from the last census data (Uganda, 2002; DRC, 1983; Togo, 2009; Guinea, 1996; Cote d’Ivoire, 1998; Mozambique, 2007), updated each year by district health officers based on estimated national annual population growth rates.

### Culture confirmation

National public health laboratories in each country performed culture confirmation of suspected cases. Cholera polymerase chain reaction (PCR) testing was not available in any of the included countries. We aimed to collect whole stool or rectal swabs from all suspected cases. In practice, the proportion of cases with a collected stool varied according to context. During large outbreaks when laboratory capacity could become overwhelmed, local staff were advised to collect the first ten cases per day only. Samples were transported in Cary-Blair transport medium to the country national reference laboratories. There, they were enriched in alkaline peptone water and plated on thiosulfate-citrate-bile-salt-sucrose (TCBS) agar. Characteristic yellow colonies were sub-cultured in non-selective medium. Resulting colonies were tested for oxidase and, if positive, considered confirmed and serogrouped. External quality control was performed by the National Institute of Communicable Diseases in South Africa using PCR.

### Rainy season definition

We adopted the definition of rainy season from the World Bank climate portal (sdwebx.worldbank.org/climateportal; accessed 2013) as follows: Uganda, Mar–Jun and Sept–Nov; Goma, DRC, Jan–May and Sept–Dec; Kinshasa, DRC, Jan–May and Oct–December; Mozambique, Oct–Mar; Cote d’Ivoire, May–Jun and Oct–Nov; Guinea (Maritime region), May–Nov; Togo (Maritime region), Apr–Jul and Sept–Nov.

### Indicators and statistical analysis

Suspected and confirmed cholera cases were summed by age group, sex, occurrence during the rainy season and clinical symptoms. We calculated the crude and corrected incidence rates for confirmed cases. Correction was done as follows: 1) for lack of culture testing, we extrapolated the proportion of culture positive results among suspect cases tested by culture to all notified suspect cases in each geographical area; 2) because culture has a sensitivity of 66% (compared to combined results from culture, dipstick, direct fluorescent antibody, multiplex-PCR and *Vibrio cholerae* O1 El Tor specific lytic phage on plaque assay as gold standard) for imperfect reported culture testing, we extrapolated the number of cultures that would have been positive if culture had a sensitivity of 100% [3]. For point 2, we conducted a literature search and identified few studies that reported culture sensitivity relative to another gold standard, as culture itself has been the gold standard for years. Consequently the study by Alam et al. was used as an approximation, recognizing that the included data may not be definitive. For calculation of case fatality ratios (CFR), we included in the denominator patients admitted to a cholera treatment center with cholera symptoms and as the numerator all deaths that were identified at the treatment center Comparisons between groups were performed using Pearson’s chi-square test. Graphs were produced with R open-access software. Statistical analyses were performed using STATA software (version 12.1, College Station, Texas 77845 USA).

### Ethical statement

Africhol provided technical and financial resources to national MoHs to support cholera surveillance. Cholera is part of the national public health surveillance through the integrated disease surveillance and response system supported by WHO. The Africhol protocol was approved and implemented by the MoH of each country. The Togolese government further elected to submit the protocol for approval to a local Togolese institutional review board (IRB). The remaining countries did not seek IRB approval as they considered that they were conducting epidemic disease surveillance and response. covered by national public health laws as an integral part of the public health mandate of the MoH and associated executing agencies.

## Results

**Fig 1:**
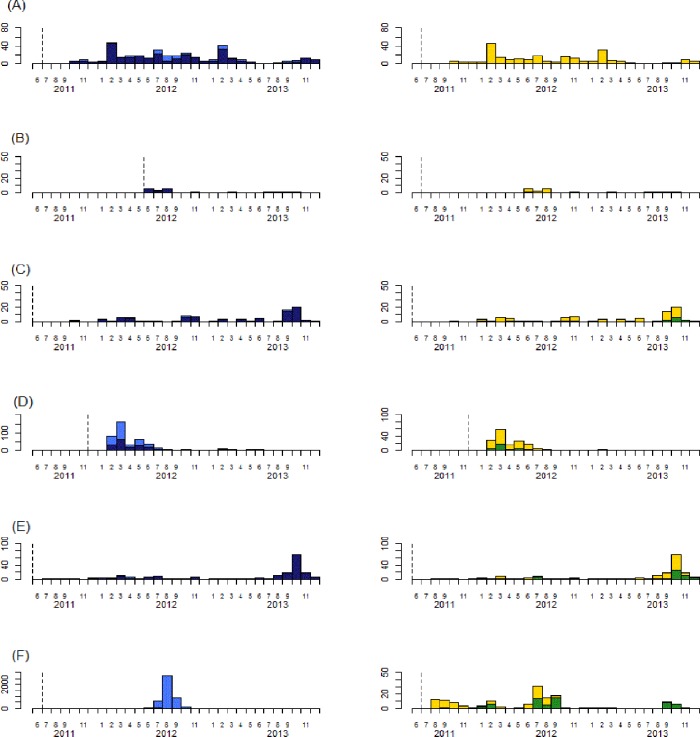
Suspect cases with culture done and Suspect cases with positive culture for *Vibrio cholera* (first part). Surveillance zone, Beira city, Mozambique (A); surveillance zone, Koumassi-Vridi-Port Boët district, Abidjan, Cote d’Ivoire (B); surveillance zone, Lake district, Togo (C); surveillance zone, Mbale-Manafwa-Butaleja districts, Uganda (D); surveillance zone, Lome-Golfe districts, Togo (E); surveillance zone, Conakry, Guinea (F); Dark blue bars show cases with culture test done, light blue bars show cases with culture test not done, green bars show cases with *Vibrio cholera* identified by culture, and yellow bars show cases having a culture negative for *Vibrio cholera*. The dashed line shows the first month of the enhanced Africhol surveillance. The dotted line shows the last month of the enhanced Africhol surveillance.

**Fig 2:**
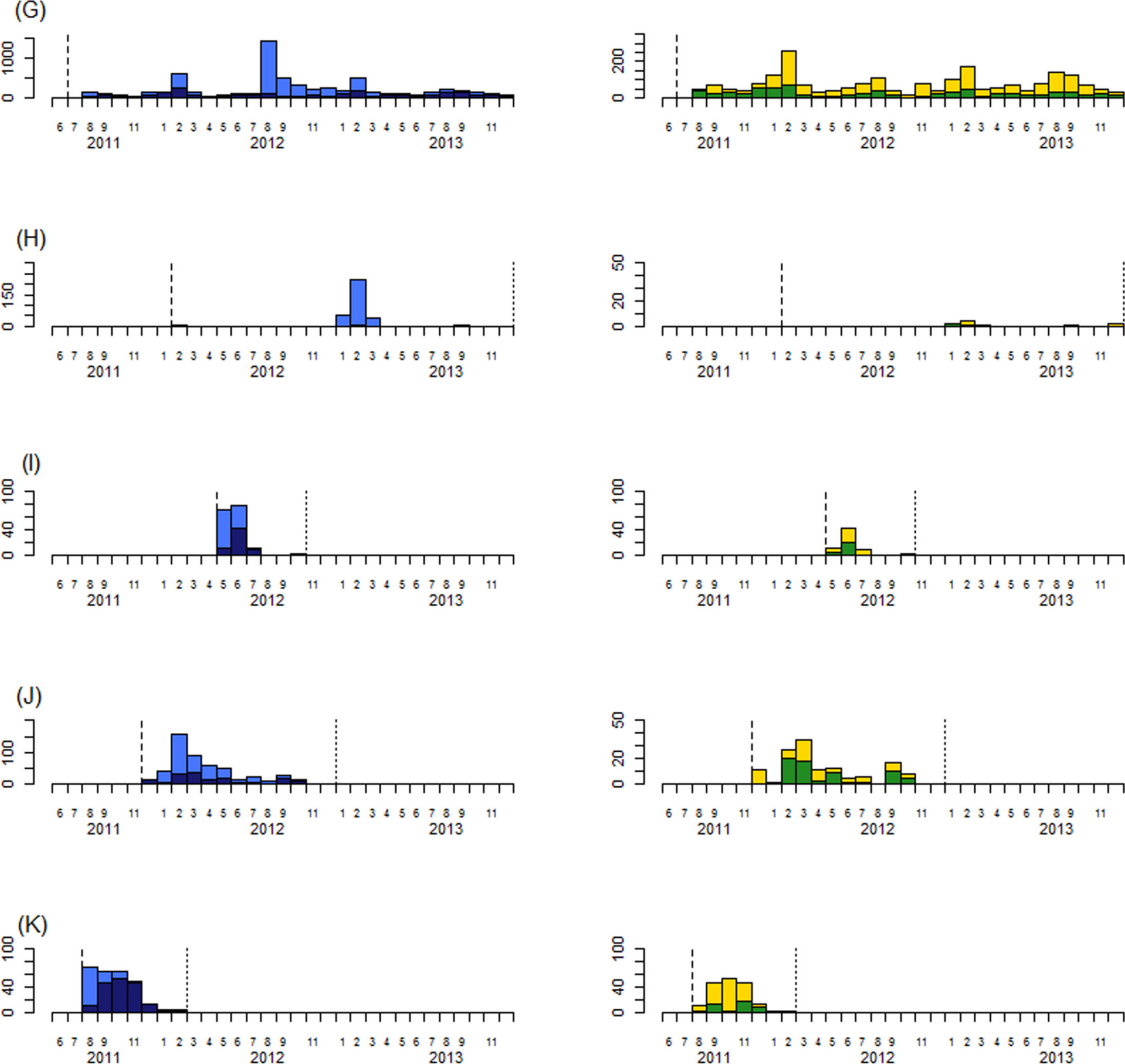
Suspect cases with culture done and Suspect cases with positive culture for *Vibrio cholera* (second part). Surveillance zone, Goma-Karisimbi districts, DRC (G); outbreak site, Pemba city, Mozambique (H); outbreak site, Adiake prefecture, Cote d’Ivoire (I); outbreak site, Kasese district, Uganda (J); outbreak site, Maluku-Kingabwa-Massina districts, Kinshasa, DRC (K). Dark blue bars show cases with culture test done, light blue bars show cases with culture test not done, green bars show cases with *Vibrio cholera* identified by culture, and yellow bars show cases having a culture negative for *Vibrio cholera*. The dashed line shows the first month of the enhanced Africhol surveillance. The dotted line shows the last month of the enhanced Africhol surveillance.

From June 2011 to December 2013, 13,377 suspect cholera case were notified: 47% (6343) occurred in surveillance zones in Goma, DRC and 34% (4585) in Conakry, Guinea *(Table 1)*. We tested 26% (3536) of all suspected cases by culture, a figure that increased to 49% when excluding zones in Goma and Conakry, which both experienced large outbreaks in August 2012 and which respectively had testing only 7.4% and 0.5% of cases during this period *(Fig 1 and Fig 2)*.

In the surveillance zones, a median of 31% of cases were culture positive ranging from 37% in Conakry, Guinea to 0% in Beira, Mozambique *(Table 1)*.

**Table 1:** Suspect cases with stool sample collected, culture test done and culture positive result. N = 13,377.

Country	Sites	Suspect cases	Stool sample collected	Tested by culture	Culture positive
		N	n (% of suspect cases)	n (% of suspect cases)	n (% of culture tested)
**Enhanced surveillance zones**					
Mozambique	Beira city	350	350 (100.0)	284 (81.1)	0 (0)
Ivory coast	KPBV, Abidjan	19	19 (100)	18 (94.7)	1 (5.6)
Guinea	Conakry city*	4585	333 (7.3)	152 (3.3)	55 (36.7)
Uganda	Mbale-Manafwa-Butaleja	400	248 (62.0)	171 (42.8)	32 (18.7)
DRC	Goma -Karisimbi**	6343	2401 (37.9)	2185 (34.4)	713 (32.6)
Togo	Lome-Golfe	216	214 (99.1)	200 (92.6)	58 (29.0)
Togo	Lake district	90	86 (95.6)	83 (92.2)	14 (16.9)
**Outbreak sites**					
Uganda	Kasese district	583	281 (48·2)	189 (32.4)	71 (37.6)
Mozambique	Pemba city	326	44 (13.5)	10 (3.1)	3 (30.0)
Ivory coast	Adiake prefecture***	161	63 (39.1)	63 (45.7)	23 (36.5)
DRC	Three districts of Kinshasa	304	297 (97.7)	181 (59.7)	49 (26.9)

Data are n (%). Suspect cases are those notified in the national surveillance system.

*4585 cases were notified during this period of time in Conakry city.

**6343 cases were notified during this period of time in Goma and Karisimbi districts.

***161 cases were notified during this period of time in Adiake prefecture.

With the exception of Adiake prefecture in Cote d’Ivoire, suspected cases were equally distributed by sex *(Table 2)*.

**Table 2:** Gender, age and season of occurrence of cholera suspect cases. N = 6280.

Country	Sites	Suspect Cases, N		Male	<5 years	5–14 y	15-59y	60 y +	Rainy season
**Enhanced surveillance zones**									
Mozambique	Beira city	350	%	55.7	39.9	19.7	37.6	2.3	63.1
			n/N	195/350	138/346	68/346	130/346	8/346	221/350
Ivory coast	KPBV, Abidjan	19	%	47.4	0	10.5	73.7	15.8	36.8
			n/N	9/19	0/19	2/19	14/19	3/19	7/19
Guinea	Conakry city	1348	%	50.7	5.6	8.6	76.9	8.9	97.7
			n/N	671/1324	72/1284	111/1284	987/1284	114/1284	1317/1348
Uganda	Mbale-Manafwa-Butaleja	400	%	50.3	10.6	20.8	60.5	8.1	78.3
			n/N	198/394	41/385	80/385	233/385	31/385	313/400
DRC	Goma -Karisimbi	2581	%	48.4	26.7	28.8	39.3	5.2	77.0
			n/N	1243/2566	688/2579	742/2579	1014/2581	135/2581	1986/2581
Togo	Lome-Golfe	216	%	56.5	7.9	15.7	72.2	4.2	74.1
			n/N	122/216	17/216	34/216	156/216	9/216	160/216
Togo	Lake district	90	%	45.6	15.7	9.0	68.5	6.7	82.2
			n/N	41/90	14/89	8/89	61/89	6/89	74/90
**Outbreak sites**									
Uganda	Kasese district	583	%	49.9	12.7	26.0	51.6	5.2	63.1
			n/N	290/581	74/581	151/581	300/581	56/581	368/583
Mozambique	Pemba city	326	%	52.5	1.0	23.0	70.9	5.1	99.1
			n/N	170/326	3/313	72/313	222/313	16/313	323/326
Ivory coast	Adiake prefecture	63	%	66.7	9.7	21.0	66.1	3.2	87.3
			n/N	42/63	6/62	13/62	41/62	2/62	55/63
DRC	Three districts of Kinshasa	304	%	46.5	10.9	17.1	66.9	5.3	44.7
			n/N	141/303	33/304	52/304	203/304	16/304	136/304

However, confirmed cases were more likely to be male *(Table 3)*. The proportion of suspected cases aged under five years ranged from zero percent in surveillance zones in Abidjan, Cote d’Ivoire to 40% in Beira, Mozambique *(Table 2);* for confirmed cases, the proportion aged under five years peaked at 29% in Goma, DRC. From 45–99% of suspected and 70–100% of confirmed cases occurred during the rainy season *(Tables 2 and 3)*.

**Table 3:** Gender, age and season of occurrence of culture confirmed cholera cases. N = 1019.

Country	Sites	Confirmed Cases, N		Male	<5 years	5–14 y	15-59y	60 y +	Rainy season
**Enhanced surveillance zones**									
Ivory coast	KPBV, Abidjan	1	%	0			100		100
			n/N	0/1			1/1		1/1
Guinea	Conakry city	55	%	55.6	12.7	21.8	61.8	3.6	87.3
			n/N	30/54	7/55	12/55	34/55	2/55	48/55
Uganda	Mbale-Manafwa-Butaleja	32	%	50.0	9.4	25.0	59.4	6.3	84.4
			n/N	16/32	3/32	8/32	19/32	2/32	27/32
DRC	Goma -Karisimbi	713	%	51.8	28.8	32.1	35.9	3.2	74.9
			n/N	368/711	205/713	229/713	256/713	23/713	534/713
Togo	Lome-Golfe	58	%	69.0	6.9	13.8	75.9	3.5	81.0
			n/N	40/58	4/58	8/58	44/58	2/58	47/58
Togo	Lake district	14	%	64.3	0	14.3	85.7	0	85.7
			n/N	9/14		2/14	12/14		12/14
**Outbreak sites**									
Uganda	Kasese district	71	%	49.3	15.5	29.6	43.7	11.3	70.4
			n/N	35/71	11/71	21/71	31/71	8/71	50/71
Mozambique	Pemba city	3	%	100	0	100	0	0	100
			n/N	3/3		3/3			3/3
Ivory coast	Adiake prefecture	23	%	65.2	8.7	26.1	65.2	0	100
			n/N	15/23	2/23	6/23	15/23		23/23
DRC	Three districts of Kinshasa	49	%	54.2	18.4	26.5	49.0	6.1	65.3
			n/N	26/48	9/49	13/49	24/49	3/49	32/49

Confirmed cases are those reported in the Africhol enhanced surveillance system

The monthly distribution of cases in Goma-Karisimbi districts (DRC), Mbale-Manafwa-Butaleja districts (Uganda), Lome and Golfe districts (Togo), Kasese district (Uganda) and Maluku-Kingabwa-Massina districts (Kinshasa, DRC) showed that cases with *Vibrio cholerae* identified by culture can be observed before the rainy season starts *(Figs 1 and 2)*.

The mean proportion of persons presenting with watery diarrhea at each site was 91% (SD 7%) and 82% (SD 16%) had vomiting. The percentage presenting with rice water stool varied from <1% to 86% and with dehydration from 33% to 99% *(Table 4).*

**Table 4:** Clinical characteristics of suspect cholera cases. N = 6280.

Country	Sites	Suspect cases, N		Watery diarrhea	Rice water stool	Vomiting	Dehydration	Confirmed cases*
**Enhanced surveillance zones**								
Mozambique	Beira city	350	%	90.1	8.7	52.7	42.6	0
			n/N	308/342	28/323	177/336	144/338	0/284
Ivory coast	KPBV, Abidjan	19	%	94.7	52.6	94.1	70.6	5.6
			n/N	18/19	10/19	16/17	12/17	1/18
Guinea	Conakry city	1348	%	94.0	74.6	88.7	34.6	36.7
			n/N	1248/1328	971/1301	1150/1297	445/1288	55/152
Uganda	Mbale-Manafwa-Butaleja	400	%	71.7	28.4	82.9	89.1	18.7
			n/N	263/367	71/250	194/234	172/193	32/171
DRC	Goma -Karisimbi	2581	%	96.9	85.6	90.5	99.1	32.6
			n/N	2498/2579	2194/2564	2320/2565	2523/2546	713/2185
Togo	Lome-Golfe	216	%	91.1	30.5	73.2	42.9	29.0
			n/N	195/214	64/210	156/213	90/210	58/200
Togo	Lake district	90	%	95.6	29.1	55.3	33.3	16.9
			n/N	86/90	25/86	47/85	29/87	14/83
**Outbreak sites**								
Uganda	Kasese district	583	%	95.2	72.0	96.8	98.4	37.6
			n/N	533/560	402/558	396/409	418/425	71/189
Mozambique	Pemba city	326	%	92.5	0.3	98.3	99.6	30.0
			n/N	297/321	1/318	287/292	268/269	3/10
Ivory coast	Adiake prefecture	63	%	84.5	13.7	88.0	46.7	36.5
			n/N	49/58	7/51	22/25	7/15	23/63
DRC	Three districts of Kinshasa	304	%	92.8	69.1	81.6	66.8	26.9
			n/N	282/304	210/304	244/299	175/262	49/181

Suspect cases are those reported in the Africhol enhanced surveillance system (n = 6280).

*Confirmed cases are those reported in the Africhol enhanced surveillance system (n = 3536).

We identified three epidemiological patterns *(Figs 1 and 2)*. In surveillance zones in Goma (DRC), confirmed cases were seen continuously throughout the surveillance period. In zones in Lome (Togo), Mbale (Uganda) and Conakry (Guinea), there were sporadic confirmed cases plus additional outbreaks at irregular intervals. Lastly, in Beira, Mozambique and Abidjan, Cote d’Ivoire, there was a history of recurrent cholera epidemics in the period leading up to Africhol implementation but as of the end of 2013, no confirmed cases had been identified for 30 months and 17 months, respectively.

Annual confirmed incidence of cholera presenting to a treatment facility per 10,000 population was <0.5 in surveillance zones, except in Goma where it was 4.6. Goma and Conakry had corrected incidences of 20.2 and 5.8 respectively, while the remaining surveillance zones had a median corrected incidence of 0.3. During outbreaks, the annualized confirmed incidence of cholera presenting to a treatment facility ranged from 0.3–3.3 and corrected incidence from 2.6 to 13.0 per 10,000 population *(Table 5)*. The ratio of the mean annual corrected incidence of confirmed cholera to the incidence of suspected cholera varied from 0.1 in Abidjan to 0.6 in Conakry while it was of 0.5 (SD 0.1) in outbreak sites.

**Table 5:** Mean annual incidence rate of cholera suspect cases and culture confirmed cases (crude or corrected) in the Africhol sites. N = 13,377.

				Suspect cases		Confirmed cases				
Country	Sites	Years$	Pop	N	Mean I suspect*	N	Mean I confirmed *	Mean corrected I (1)	Mean corrected I (2)	Ratio I (2) / I suspect
**Enhanced Surveillance zones**										
Mozambique	Beira city	2.4	431,583	350	3.4	0	0	0	0	-
Ivory coast	KPBV, Abidjan	1.6	820,203	19	0.2	1	0.0	0.0	0.0	0.1
Guinea	Conakry city	2.5	173,970	4585	10.5	55	0.1	3.8	5.8	0.6
Uganda	Mbale-Manafwa-Butaleja	2.1	1,030,200	400	4.4	32	0.3	0.8	1.2	0.3
DRC	Goma–Karisimbi	2.8	569,183	6343	40.5	713	4.6	13.2	20.2	0.5
Togo	Lome-Golfe	2.8	885,679	216	0.9	58	0.2	0.3	0.4	0.4
Togo	Lake district	2.8	775,063	90	0.4	14	0.1	0.1	0.1	0.3
**Outbreak sites**										
Uganda	Kasese district	1.1	770,000	583	6.8	71	0.8	2.6	3.9	0.6
Mozambique	Pemba city	0.9	121,967	326	28.4	3	0.3	8.5	13.0	0.5
Ivory coast	Adiake prefecture	0.5	151,651	161	23.1	23	3.3	8.4	12.8	0.6
DRC	Three districts of Kinshasa	0.6	782,631	304	6.2	49	1.0	1.7	2.6	0.4

Suspect cases are those notified in the national surveillance system. Confirmed cases are those reported in the Africhol surveillance system.

$Duration of the follow-up period.

* Mean Incidence (cases /10,000 pop/ year).

(1) Mean corrected incidence (cases /10,000 pop/ year) for lack of testing. Correction on lack of testing was done for each sites according to the figures provided in Table 1.

(2) Mean corrected incidence (cases /10,000 pop/ year) for lack of testing AND culture sensitivity. Correction on culture sensitivity was done according to the figure provided in the methods part.

Of 5980 suspected cases identified in a treatment facility with a documented outcome, 69 died. The median CFR was 1.1% [IQR: 0.7–4.3]. The CFR varied from zero percent in Abidjan, Cote d’Ivoire to 10% in Lake district, Togo *(Table 6)*. We found no statistical differences in the CFR between confirmed and non-confirmed cases. However we observed that deceased patients were less likely to have received culture testing than those alive at discharge (35.3% vs. 55.6%, chi-square p. value = 0.001).

**Table 6:** Case fatality ratio (CFR) among suspected and culture confirmed cholera cases. N = 5980.

		Suspect cases*			Culture confirmed cases*		
Country	Sites	N	Deaths	CFR (%)	N	Deaths	CFR (%)
**Enhanced surveillance zone**							
Mozambique	Beira city	350	2	0.6	0	0	0
Ivory coast	KPBV, Abidjan	19	0	0.0	1	0	0
Guinea	Conakry city	1297	15	1.2	48	2	4.2
Uganda	Mbale-Manafwa-Butaleja	400	18	4.5	32	1	3.1
DRC	Goma -Karisimbi	2491	4	0.2	682	1	0.2
Togo	Lome-Golfe	132	1	0.8	58	0	0
Togo	Lake district	48	4	8.3	10	1	10.0
**Outbreak sites**							
Uganda	Kasese district	583	8	1.4	71	0	0
Mozambique	Pemba city	326	3	0.9	3	0	0
Ivory coast	Adiake prefecture	60	3	5.0	23	2	8.7
DRC	Three districts of Kinshasa	274	11	4.0	43	1	2.3

*Suspect cases and culture confirmed cases are those having a documented “patient outcome” in the Africhol surveillance system.

## Discussion

In the Africhol surveillance zones, we found an overall annual corrected incidence of confirmed cholera presenting to a treatment facility of 0.3 cases per 10,000 population, which increased to 20 cases per 10,000 during large epidemics. Strong spatial and temporal clustering occurred, with most cases from surveillance zones in Conakry, Guinea and Goma, DRC. Within our study many suspected cases were not cholera confirmed by culture. Furthermore the CRF measured at clinic level remained low in our surveillance sites. From the surveillance data collected in our sites, we were able to identify three epidemiological patterns of cholera: confirmed cases throughout the year such as Goma (DRC); sporadic cases plus additional outbreaks at irregular intervals such as in Lome (Togo), Mbale (Uganda), and Conakry (Guinea); and history of recurrent cholera epidemics but no cases during the surveillance period, such as Beira (Mozambique) or Abidjan (Cote d’Ivoire). Whatever the location, we found that most cholera cases occurred during the rainy season.

Our incidence estimates for confirmed cases showed similar fluctuations by place and time as those reported previously for suspected cases but are substantially lower than estimates modeled from WHO mortality strata [4–14]. In most national cholera surveillance systems, etiologic confirmation occurs only for the first suspected cases, before outbreak declaration. Subsequently, any person with acute watery diarrhea usually would be reported as a cholera case, even though some of these will have other etiologies. Consequently, syndromic surveillance–as reported by most previous studies–likely overestimates cholera incidence.

Moreover, the proportion of culture confirmed cases varied widely by site emphasizing the utility of laboratory based studies. At the extreme, in Beira, Mozambique, where a history of large outbreaks likely led providers to have a high index of suspicion for cholera, all sampled suspected cases remained negative for *V*. *cholera* [11].

The wide variation we found may have resulted from differences in health care seeking behavior, health care access, type and extent of available health structures, health work training, and adherence to case definitions. For instance, treatment centers in Goma, DRC provided care for patients with any diarrheal disease regardless of etiology, did not charge fees, and treated persons of all ages. In other Africhol sites, cholera treatment centers offering free treatment were established only when authorities declared the outbreak. These issues also may have led to the differences in health care access behaviors and therefore to clinical presentation across sites. Other factors may lead to underestimation of incidence. For example, not all patients will present for care at a medical facility and data collection and reporting may be incomplete. However, our system was not designed to assess these issues.

While our incidence rates were lower than those from early reports, CFRs for confirmed cholera cases were consistent with those for suspect cases attending health facilities [5,11]. The low identified CFRs emphasize the great strides some cholera endemic countries have made in identifying outbreaks rapidly and improving clinical management. They might also reflect the sensitization of populations in high-risk areas to the importance of seeking timely medical care. Our CFR estimates were limited by our inability to assess deaths in the community which contribute to potential underestimation. Lastly, both our CFRs and overall incidence rates were limited by lack of active community-based surveillance, an objective for which our work was not funded. It is likely that this problem was particularly large for deaths: for example, a study from Kenya found that most deaths occurred among persons who had not sought treatment [15]. Future geographically focused studies might address this issue. In theory, health utilization surveys and capture-recapture analysis could help with estimation of surveillance system sensitivity. However, in epidemic cholera prone settings in Africa, health care utilization surveys are seldom appropriate given the lack of human resources relative to the immediate priority of outbreak control. Capture-recapture analyses similarly are not feasible, given the fluid nature of a surveillance system in which cholera treatment centers are established and dismantled relative to cholera case counts.

We identified three epidemiological patterns of cholera in our sites: those with confirmed cases throughout the year such as Goma (DRC); those with sporadic cases plus additional outbreaks at irregular intervals such as in Lome (Togo), Mbale (Uganda), and Conakry (Guinea); and those with a history of recurrent cholera epidemics but no cases during the surveillance period, such as Beira (Mozambique) or Abidjan (Cote d’Ivoire). The presence of sporadic cases without ensuing outbreaks may occur from occasional introduction of infected persons into a low risk community, e.g., a community with recent cholera and a high degree of population immunity or a community with good water and sanitation infrastructure. By contrast, sustained occurrence of confirmed cases may result from ongoing environmental source contamination from which a continuously renewed susceptible, non-immune population is infected; this may have occurred in Goma, which has experienced several waves of immigration due to regional conflicts.

We found that most cholera cases occurred during the rainy season. However the presence of cases before the rain start suggests that the rainy season may play a role of outbreak amplificatory. Previous studies have found similar results [16]. Substantial precipitation can cause flooding and subsequent mixing of drinking water (pond, well, lake, river) with sewage in areas with poor sanitation [17]. Alternatively, the rainy season may trigger human movement, such as the seasonal migration of fishermen along the West African coast or in interior lakes [16,18–20].

Our study had several limitations other than those mentioned above. We report data from only eleven geographical sites located in six countries and this may not be generalizable to other African settings. Our correction of incidence based on the lack of testing was applied uniformly across the surveillance period without taking into account seasonal variations. We used a single value to correct for culture sensitivity although culture results may vary by setting based on factors such as laboratory technician skills and stool collection and transportation methods. Finally, CFRs were difficult to assess for confirmed cholera cases because of lack of testing.

In the African cholera context, oral cholera vaccine may provide an important short- and medium-term prevention and control measure in addition to case management and long-term efforts to improve water, sanitation and hygiene (WaSH Despite the utility of mass OCV campaigns have been already demonstrated in some African areas, it remains difficult to determine the best strategy to use and if a relatively circumscribed immunization campaign can prevent an epidemic on the scale of Zimbabwe or Haiti [21–25]. Short duration and geographically focal outbreaks as described in our results will make reactive OCV use challenging, as it was the case in Mozambique [11]. Even in settings with large outbreaks such as Goma or Conakry, cases may occur over a brief period in relatively small geographic areas, such as districts. Preventive immunization may be indeed more appropriate to reduce cholera in target communities, with a potential secondary benefit of reducing transmission outside the target zone… We might also learn from *Neisseria meningitidis* (Nm) meningitis in the meningitis belt [26]. As with cholera, Nm outbreaks were often highly focal, of short duration, difficult to predict, and occurred in areas with limited laboratory facilities. The strategy for years was reactive campaigns following notification of an epidemic. However, vaccine frequently arrived after the epidemic peak and thus its overall efficacy questioned. This situation changed with the introduction of a low-cost Nm serogroup A conjugate vaccine (MenAfriVac) through national preventive immunization programs via mass campaigns into persons 1 to 29 years of age [27]. The analogy between OCV and MenAfriVac is also based on the need for national and international commitment for an evidence-based prevention strategy, availability of low-cost vaccine produced in sufficient quantity, and the availability of adequate financial and human resources.

While limited to health care facilities, our study presents some of the only prospectively obtained incidence data currently available for Africa. Our findings suggest that confirmed cholera burden is substantially lower than that reported from previous studies based on suspected cholera cases, and that incidence varies substantially over time and place. Efficient use of resources, such as vaccines, could be enhanced by better definition of cholera hot-spots, community behaviors that contribute to cholera spread, and high risk populations, particularly those likely to contribute to seasonal cholera spread.

Because of the frequent occurrence of non-cholera causes of diarrhea in cholera endemic zones, development of public health strategies would benefit from reinforcement of local laboratory capacities for diagnosing *Vibrio cholerae*, something that also would benefit from development of better low-cost diagnostic methods. Environmental reservoirs should be identified and mitigation strategies developed. Determination of other diarrheal disease etiologies across all age groups will help determine the utility of etiology specific interventions. OCV interventions must be conducted, monitored and evaluated to better assess their cost-effectiveness and their health impact among at-risk populations in African contexts. Finally, there is a role for evaluation of low-cost water and sanitation improvements within an integrated strategy for cholera prevention and control.

## Supporting Information

S1 Checklist STROBE checklist. (PDF)
