# Supplementary material for: Cholera Incidence and Mortality in Sub-Saharan African Sites during Multi-country Surveillance
Source: PLoS Negl Trop Dis. 2016 May 17;10(5):e0004679. doi: 10.1371/journal.pntd.0004679 (PMC4871502; doi:10.1371/journal.pntd.0004679)
Supplement: S1 Checklist — (PDF) [file pntd.0004679.s001.pdf]

STROBE Statement—checklist of items that should be included in reports of observational studies

	Item No	Recommendation
**Title and abstract**	1	Indicate the study's design with a commonly used term in the title or the abstract **[Within the title page 1 and method section of the abstract page 2]**<br><br>Cholera incidence and mortality in sub-Saharan African sites during multi-country surveillance
		(*b*) Provide in the abstract an informative and balanced summary of what was done and what was found **[See results section of abstract page 2]**
**Introduction**		
Background/rationale	2	Explain the scientific background and rationale for the investigation being reported **[page 4]**<br>From 2007 to 2012, at least 20 African countries reported more than 100,000 cases of cholera (World Health Organization (WHO) weekly epidemiological records, 2007–2012). However the surveillance of cholera has weaknesses. Reporting is non-exhaustive for various reasons such as individual and community fears of stigmatization and economic loss. Reporting from district to national levels may be delayed or incomplete. According to WHO, only 3% to 5% of all cases are laboratory confirmed. A variety of case definitions are used across countries, which could lead to cholera over or under-reporting. Finally, few countries have implemented case-based surveillance, with information at national level provided in the form of weekly summaries limited to cumulative case numbers and deaths.
Objectives	3	State specific objectives, including any prespecified hypotheses<br>**[page 4]**<br>We present cholera incidence results and the associated case fatality ratio from eleven geographical zones located in six Africhol countries having the strongest performing surveillance systems.
**Methods**		
Study design	4	Present key elements of study design early in the paper<br>**[page 4]**<br>We conducted a population -based cholera surveillance in all cholera treatment facilities in given geographic zones.
Setting	5	Describe the setting, locations, and relevant dates, including periods of recruitment, exposure, follow-up, and data collection<br>**[pages 5-6]**<br>We included eight enhanced surveillance zones located in areas of known recent cholera occurrence were included in the analysis. Their location and starting dates were as follows: 1) Togo: five districts of Lome and Golfe district, Jun 2011; 2) Togo: Lake district in the Maritime region, Jun 2011; 3) Democratic Republic of Congo (DRC): Goma and Karisimbi districts, Aug 2011; 4) Guinea: five districts of Conakry, Jul 2011; 5) Uganda: Manafwa, Mbale, and Butaleja districts, Dec 2011; 6) Mozambique: Beira city, Aug 2011; 7) Cote d'Ivoire: one district of Abidjan, Koumassi–Port Bouet–Vridi district (KPBV), Jun 2012. While data collection is currently ongoing, here we include only surveillance data collected through Dec 31st, 2013. We included also outbreaks sites in Kasese district, Uganda (Oct 2011–Dec 2012); Pemba city, Mozambique (Jan 2013–Dec 2013); Adiake prefecture, Cote d'Ivoire (May–Oct 2012); and three districts of Kinshasa (Maluku, Kingabwa , and Massina districts), DRC (Jul 2011–Feb 2012). Within specifically defined study zones, we included all health care facilities known to treat cholera cases as following: 1) Conakry, Guinea: The infectious diseases and paediatric departments of Donka hospital, the additional cholera treatment center (CTC) in the Ratoma neighbourhood opened during the 2012 epidemic was also included. 2) Lome, Togo: The infectious

		disease and paediatric departments of the Centre Hospitalier Universitaire, Be Hospital, and other district health centres in which a temporary cholera treatment center was opened. 3) Lake District, Togo: The infectious diseases and paediatric departments of Aneho Hospital and health centres with temporary treatment centers. 4) Goma-Karisimbi district, DRC: The cholera treatment centers located in the General Provincial Hospital, the Buhimba cholera treatment and the Kiziba temporary cholera treatment unit. 5) Maluku-Kingabwa-Massina district, Kinshasa, DRC: The cholera treatment centers of Kingabwa and Malaku and the cholera treatment unit of Massina. 6) Abidjan, Koumassi-Port Bouet, Vridi District, Cote d'Ivoire: The infectious diseases and paediatric departments of Port Bouet and Koumassi Hospitals and the temporary cholera treatment center at the Vridi Health Centre. 7) Adiake prefecture, Cote d'Ivoire: Adiake general hospital and additional temporary treatment centers. 8) Mbale-Manafwa-Buteleja district, Uganda: Nabiganda health center, Namatela health center and Busiu health center. 9) Kasese district, Uganda. Bwera hospital, Kayangi health center, Kagando hospital, Kinyamaseke health center, Kitholhu health center and other temporary treatment centers. 10) Beira, Mozambique: Ponta-Gea health center, Macurrungo health center, Munhava health center, Macurrungo and the central hospital of Beira. 11) Pemba city, Mozambique: The cholera treatment center of Pemba city.
	6	*Cohort study*—Give the eligibility criteria, and the sources and methods of selection of participants. Describe methods of follow-up.<br>**[page 5]**<br>Patients were followed in all the cholera treatment facilities of a given surveillance area. In area without known ongoing cholera, a suspected cholera case was defined as a patient aged two years or more that developed severe dehydration or died from acute watery diarrhea. In areas with known cholera, a suspected case was defined as a patient aged two years or more that developed acute watery diarrhea, with or without vomiting. A confirmed case was defined as a suspect cholera having a stool culture positive for *Vibrio cholera*.<br><br>*Case-control study*—Give the eligibility criteria, and the sources and methods of case ascertainment and control selection. Give the rationale for the choice of cases and controls **[N/A]**<br>*Cross-sectional study*—Give the eligibility criteria, and the sources and methods of selection of participants **[N/A]**
		(*b*) *Cohort study*—For matched studies, give matching criteria and number of exposed and unexposed **[N/A]**<br>*Case-control study*—For matched studies, give matching criteria and the number of controls per case **[N/A]**
Variables	7	Clearly define all outcomes, exposures, predictors, potential confounders, and effect modifiers. Give diagnostic criteria, if applicable<br>**[page 7]**<br>We presented the proportions of suspected and confirmed cholera cases by age group, sex, occurrence during the rainy season and clinical symptoms. We calculated the crude incidence rate of cholera and corrected incidence rates for confirmed cases. We also presented case fatality ratios for suspected and confirmed cholera.
Data sources/ measurement	8*	For each variable of interest, give sources of data and details of methods of assessment (measurement). Describe comparability of assessment methods if there is more than one group<br>**[pages 6-7]**<br>In the enhanced surveillance zones and outbreak sites, the MoH teams collected data at health centers level using the same standardized data collection forms, which included sex, age, location, date of symptoms, culture results but also clinical information such as watery diarrhea, rice water stool, vomiting, dehydration. We identified all deaths among patients admitted to a cholera treatment facility. We did not include deaths occurring in the community or after treatment center discharge. In parallel, the MoH continued to register the overall number of suspected cases in their routine surveillance system using line lists with a limited number of variables (date of

		onset, district, age and sex). We used district–level population estimates for 2011 or 2012 that corresponded to the geographic area under surveillance. The 2011 and 2012 population estimates were derived from the last census data (Uganda, 2002; DRC, 1983; Togo, 2009; Guinea, 1996; Cote d'Ivoire, 1998; Mozambique, 2007), updated each year by district health officers based on estimated national annual population growth rates.<br>We adopted the definition of rainy season from the World Bank climate portal (sdwebx.worldbank.org/climateportal; accessed 2013) as follows: Uganda, Mar–Jun and Sept–Nov; Goma, DRC, Jan–May and Sept–Dec; Kinshasa, DRC, Jan–May and Oct–December*;* Mozambique, Oct–Mar; Cote d'Ivoire, May–Jun and Oct–Nov; Guinea (Maritime region), May–Nov; Togo (Maritime region), Apr–Jul and Sept–Nov.
Bias	9	Describe any efforts to address potential sources of bias<br>**[page 7]**<br>We calculated corrected incidence rates taking into account the lack of culture testing in each given surveillance zone and the lack of sensitivity of culture test.
Study size	10	Explain how the study size was arrived at<br>**[N/A]**
Quantitative variables	11	Explain how quantitative variables were handled in the analyses. If applicable, describe which groupings were chosen and why<br>**[N/A]**
Statistical methods	12	(*a*) Describe all statistical methods, including those used to control for confounding
		**[pages 7-8]**<br>We calculated the crude and corrected incidence rates for confirmed cases. Correction was done as follows: 1) for lack of culture testing, we extrapolated the proportion of culture positive results among suspect cases tested by culture to all notified suspect cases in each geographical area; 2) because culture has a sensitivity of 66% (compared to combined results from culture, dipstick, direct fluorescent antibody, multiplex-PCR and *Vibrio cholerae* O1 El Tor specific lytic phage on plaque assay as gold standard) for imperfect reported culture testing, we extrapolated the number of cultures that would have been positive if culture had a sensitivity of 100% [3]. For point 2, we conducted a literature search and identified few studies that reported culture sensitivity relative to another gold standard, as culture itself has been the gold standard for years. Consequently the study by Alam et al. was used as an approximation, recognizing that the included data may not be definitive. For calculation of case fatality ratios, we included in the denominator patients admitted to a cholera treatment center with cholera symptoms and as the numerator all deaths that were identified at the treatment center. Comparisons between groups were performed using Pearson's chi-square test. Graphs were produced with R open-access software. Statistical analyses were performed using STATA software (version 12.1, College Station, Texas 77845 USA).
		(*b*) Describe any methods used to examine subgroups and interactions<br>**[N/A]**
		(*c*) Explain how missing data were addressed<br>**[N/A]**
		(*d*) *Cohort study*—If applicable, explain how loss to follow-up was addressed **[N/A]**<br>*Case-control study*—If applicable, explain how matching of cases and controls was addressed **N/A]**<br>*Cross-sectional study*—If applicable, describe analytical methods taking account of sampling strategy **[N/A]**
		(*e*) Describe any sensitivity analyses

Results

Participants	13*	(a) Report numbers of individuals at each stage of study—eg numbers potentially eligible,

		examined for eligibility, confirmed eligible, included in the study, completing follow-up, and analysed **[N/A]**
		(b) Give reasons for non-participation at each stage **[N/A]**
		(c) Consider use of a flow diagram **[N/A]**
Descriptive data	14*	Give characteristics of study participants (eg demographic, clinical, social) and information on exposures and potential confounders<br>**[pages 8-12 and tables 1, 2a, 2b, 3]**<br>From June 2011 to December 2013, 13,377 suspect cholera cases were notified: 47% (6343) occurred in surveillance zones in Goma, DRC and 34% (4585) in Conakry, Guinea.<br>In the surveillance zones, a median of 31% of cases were culture positive ranging from 37% in Conakry, Guinea to 0% in Beira, Mozambique. With the exception of Adiake prefecture in Cote d'Ivoire, suspected cases were equally distributed by sex. The proportion of suspected cases aged under five years ranged from zero percent in surveillance zones in Abidjan, Cote d'Ivoire to 40% in Beira, Mozambique*;* for confirmed cases, the proportion aged under five years peaked at 29% in Goma, DRC.<br>From 45–99% of suspected and 70–100% of confirmed cases occurred during the rainy season. The monthly distribution of cases in Goma-Karisimbi districts (DRC), Mbale-Manafwa-Butaleja districts (Uganda), Lome and Golfe districts (Togo), Kasese district (Uganda) and Maluku-Kingabwa-Massina districts (Kinshasa, DRC) showed that cases with *Vibrio cholerae* identified by culture can be observed before the rainy season starts.<br>The mean proportion of persons presenting with watery diarrhea at each site was 91% (SD 7%) and 82% (SD 16%) had vomiting. The percentage presenting with rice water stool varied from <1% to 86% and with dehydration from 33% to 99%.
		(b) Indicate number of participants with missing data for each variable of interest<br>**[pages 8-9, tables 1, 2a, 2b, 3 and figure 1]**<br>We tested 26% (3536) of all suspected cases by culture *(table 1)*, a figure that increased to 49% when excluding zones in Goma and Conakry, which both experienced large outbreaks in August 2012 and which respectively had testing only 7.4% and 0.5% of cases during this period *(Figure 1).*
		(c) *Cohort study*—Summarise follow-up time (eg, average and total amount) [**see table 4]**
Outcome data	15*	*Cohort study*—Report numbers of outcome events or summary measures over time<br>[**pages 12-13-14, tables 4 and 5 and figure 1]**<br>Annual confirmed incidence of cholera presenting to a treatment facility per 10,000 population was <0.5 in surveillance zones, except in Goma where it was 4.6. Goma and Conakry had corrected incidences of 20.2 and 5.8 respectively, while the remaining surveillance zones had a median corrected incidence of 0.3. During outbreaks, the annualized confirmed incidence of cholera presenting to a treatment facility ranged from 0.3–3.3 and corrected incidence from 2.6 to 13.0 per 10,000 population. The ratio of the mean annual corrected incidence of confirmed cholera to the incidence of suspected cholera varied from 0.1 in Abidjan to 0.6 in Conakry while it was of 0.5 (SD 0.1) in outbreak sites.<br>Of 5980 suspected cases identified in a treatment facility with a documented outcome, 69 died. The median CFR was 1.1% [IQR: 0.7–4.3]. The CFR varied from zero percent in Abidjan, Cote d'Ivoire to 10% in Lake district, Togo. We found no statistical differences in the CFR between confirmed and non-confirmed cases. However we observed that deceased patients were less likely to have received culture testing than those alive at discharge (35.3% vs. 55.6%, chi-square p. value= 0.001).
		Case-control study—Report numbers in each exposure category, or summary measures of exposure **[N/A]**
		Cross-sectional study—Report numbers of outcome events or summary measures **[N/A]**
Main results	16	(*a*) Give unadjusted estimates and, if applicable, confounder-adjusted estimates and their precision (eg, 95% confidence interval). Make clear which confounders were adjusted for and why they were included **[N/A]**
		(*b*) Report category boundaries when continuous variables were categorized **[N/A]**
		(*c*) If relevant, consider translating estimates of relative risk into absolute risk for a meaningful time period **[N/A]**
Other analyses	17	Report other analyses done—eg analyses of subgroups and interactions, and sensitivity analyses

		**[page 12, figure 1]**<br>We identified three epidemiological patterns. In surveillance zones in Goma (DRC), confirmed cases were seen continuously throughout the surveillance period. In zones in Lome (Togo), Mbale (Uganda) and Conakry (Guinea), there were sporadic confirmed cases plus additional outbreaks at irregular intervals. Lastly, in Beira, Mozambique and Abidjan, Cote d'Ivoire, there was a history of recurrent cholera epidemics in the period leading up to Africhol implementation but as of the end of 2013, no confirmed cases had been identified for 30 months and 17 months, respectively.
**Discussion**		
Key results	18	Summarise key results with reference to study objectives<br>**[page 14]**<br>In the Africhol surveillance zones, we found an overall annual corrected incidence of confirmed cholera presenting to a treatment facility of 0.3 cases per 10,000 population, which increased to 20 cases per 10,000 during large epidemics. Strong spatial and temporal clustering occurred, with most cases from surveillance zones in Conakry, Guinea and Goma, DRC. Within our study many suspected cases were not cholera confirmed by culture. Furthermore the CRF measured at clinic level remained low in our surveillance sites. From the surveillance data collected in our sites, we were able to identify three epidemiological patterns of cholera: confirmed cases throughout the year such as Goma (DRC); sporadic cases plus additional outbreaks at irregular intervals such as in Lome (Togo), Mbale (Uganda), and Conakry (Guinea); and history of recurrent cholera epidemics but no cases during the surveillance period, such as Beira (Mozambique) or Abidjan (Cote d'Ivoire). Whatever the location, we found that most cholera cases occurred during the rainy season.
Limitations	19	Discuss limitations of the study, taking into account sources of potential bias or imprecision. Discuss both direction and magnitude of any potential bias<br>**[pages 15 and 16]**<br>The wide variation of culture confirmed cases may have resulted from differences in health care seeking behavior, health care access, type and extent of available health structures, health work training, and adherence to case definitions. For instance, treatment centers in Goma, DRC provided care for patients with any diarrheal disease regardless of etiology, did not charge fees, and treated persons of all ages. In other Africhol sites, cholera treatment centers offering free treatment were established only when authorities declared the outbreak. These issues also may have led to the differences in health care access behaviors and therefore to clinical presentation across sites. Other factors may lead to underestimation of incidence. For example, not all patients will present for care at a medical facility and data collection and reporting may be incomplete. However, our system was not designed to assess these issues.<br><br>Our CFR estimates were limited by our inability to assess deaths in the community which contribute to potential underestimation. Both our CFRs and overall incidence rates were limited by lack of active community-based surveillance, an objective for which our work was not funded. It is likely that this problem was particularly large for deaths: for example, a study from Kenya found that most deaths occurred among persons who had not sought treatment. Future geographically focused studies might address this issue. In theory, health utilization surveys and capture-recapture analysis could help with estimation of surveillance system sensitivity. However, in epidemic cholera prone settings in Africa, health care utilization surveys are seldom appropriate given the lack of human resources relative to the immediate priority of outbreak control. Capture-recapture analyses similarly are not feasible, given the fluid nature of a surveillance system in which cholera treatment centers are established and dismantled relative to cholera case counts.<br><br>Our study had several limitations other than those mentioned above. We report data from only eleven geographical sites located in six countries and this may not be generalizable to other African settings. Our correction of incidence based on the lack of testing was applied uniformly across the surveillance period without taking into account seasonal variations. We used a single value to correct for culture sensitivity although culture results may vary by setting based on factors such as laboratory technician skills and stool collection and transportation methods. Finally, CFRs were difficult to assess for confirmed cholera cases because of lack of testing.

Interpretation	20	Give a cautious overall interpretation of results considering objectives, limitations, multiplicity of analyses, results from similar studies, and other relevant evidence<br>**[pages 15-16]**<br>Our incidence estimates for confirmed cases showed similar fluctuations by place and time as those reported previously for suspected cases but are substantially lower than estimates modeled from WHO mortality strata. In most national cholera surveillance systems, etiologic confirmation occurs only for the first suspected cases, before outbreak declaration. Subsequently, any person with acute watery diarrhea usually would be reported as a cholera case, even though some of these will have other etiologies. Consequently, syndromic surveillance – as reported by most previous studies – likely overestimates cholera incidence.<br>Moreover, the proportion of culture confirmed cases varied widely by site emphasizing the utility of laboratory based studies. At the extreme, in Beira, Mozambique, where a history of large outbreaks likely led providers to have a high index of suspicion for cholera, all sampled suspected cases remained negative for *V. cholera* [11].<br>While our incidence rates were lower than those from early reports, CFRs for confirmed cholera cases were consistent with those for suspect cases attending health facilities [5,11]. The low identified CFRs emphasize the great strides some cholera endemic countries have made in identifying outbreaks rapidly and improving clinical management. They might also reflect the sensitization of populations in high-risk areas to the importance of seeking timely medical care.<br>We identified three epidemiological patterns of cholera in our sites: those with confirmed cases throughout the year such as Goma (DRC); those with sporadic cases plus additional outbreaks at irregular intervals such as in Lome (Togo), Mbale (Uganda), and Conakry (Guinea); and those with a history of recurrent cholera epidemics but no cases during the surveillance period, such as Beira (Mozambique) or Abidjan (Cote d'Ivoire). The presence of sporadic cases without ensuing outbreaks may occur from occasional introduction of infected persons into a low risk community, e.g., a community with recent cholera and a high degree of population immunity or a community with good water and sanitation infrastructure. By contrast, sustained occurrence of confirmed cases may result from ongoing environmental source contamination from which a continuously renewed susceptible, non-immune population is infected; this may have occurred in Goma, which has experienced several waves of immigration due to regional conflicts.<br>We found that most cholera cases occurred during the rainy season. However the presence of cases before the rain start suggests that the rainy season may play a role of outbreak amplificatory. Previous studies have found similar results [16]. Substantial precipitation can cause flooding and subsequent mixing of drinking water (pond, well, lake, river) with sewage in areas with poor sanitation [17]. Alternatively, the rainy season may trigger human movement, such as the seasonal migration of fishermen along the West African coast or in interior lakes [16,18–20].
Generalisability	21	Discuss the generalisability (external validity) of the study results<br>**[pages 17-18]**<br>While limited to health care facilities, our study presents some of the only prospectively obtained incidence data currently available for Africa. Our findings suggest that confirmed cholera burden is substantially lower than that reported from previous studies based on suspected cholera cases, and that incidence varies substantially over time and place. Efficient use of resources, such as vaccines, could be enhanced by better definition of cholera hot-spots, community behaviors that contribute to cholera spread, and high risk populations, particularly those likely to contribute to seasonal cholera spread.<br>Because of the frequent occurrence of non-cholera causes of diarrhea in cholera endemic zones, development of public health strategies would benefit from reinforcement of local laboratory capacities for diagnosing *Vibrio cholerae*, something that also would benefit from development of better low-cost diagnostic methods. Environmental reservoirs should be identified and mitigation strategies developed. Determination of other diarrheal disease etiologies across all age groups will help determine the utility of etiology specific interventions. OCV interventions must be conducted, monitored and evaluated to better assess their cost-effectiveness and their health impact among at-risk populations in African contexts. Finally, there is a role for evaluation of low-cost water and sanitation improvements within an integrated strategy for cholera prevention and control.
**Other information**		
Funding	22	Give the source of funding and the role of the funders for the present study and, if applicable,

for the original study on which the present article is based

Financial support was provided by the Bill & Melinda Gates Foundation through the Africhol project (grant number: OPPGH5233), administered by the Agence de Medecine Preventive (AMP), Paris, France. The funder had no role in study design, data collection and analysis, decision to publish or preparation of the manuscript.

*Give information separately for cases and controls in case-control studies and, if applicable, for exposed and unexposed groups in cohort and cross-sectional studies.

Note: An Explanation and Elaboration article discusses each checklist item and gives methodological background and published examples of transparent reporting. The STROBE checklist is best used in conjunction with this article (freely available on the Web sites of PLoS Medicine at http://www.plosmedicine.org/, Annals of Internal Medicine at http://www.annals.org/, and Epidemiology at http://www.epidem.com/). Information on the STROBE Initiative is available at www.strobe-statement.org.
